# Supplementary figures and images for: Staphylococcus aureus agr-type vs genetic background: molecular signatures determining differential metabolism and virulence potential
Source: PLoS One. 2026 Jun 3;21(6):e0350108. doi: 10.1371/journal.pone.0350108 (PMC13232812; doi:10.1371/journal.pone.0350108)

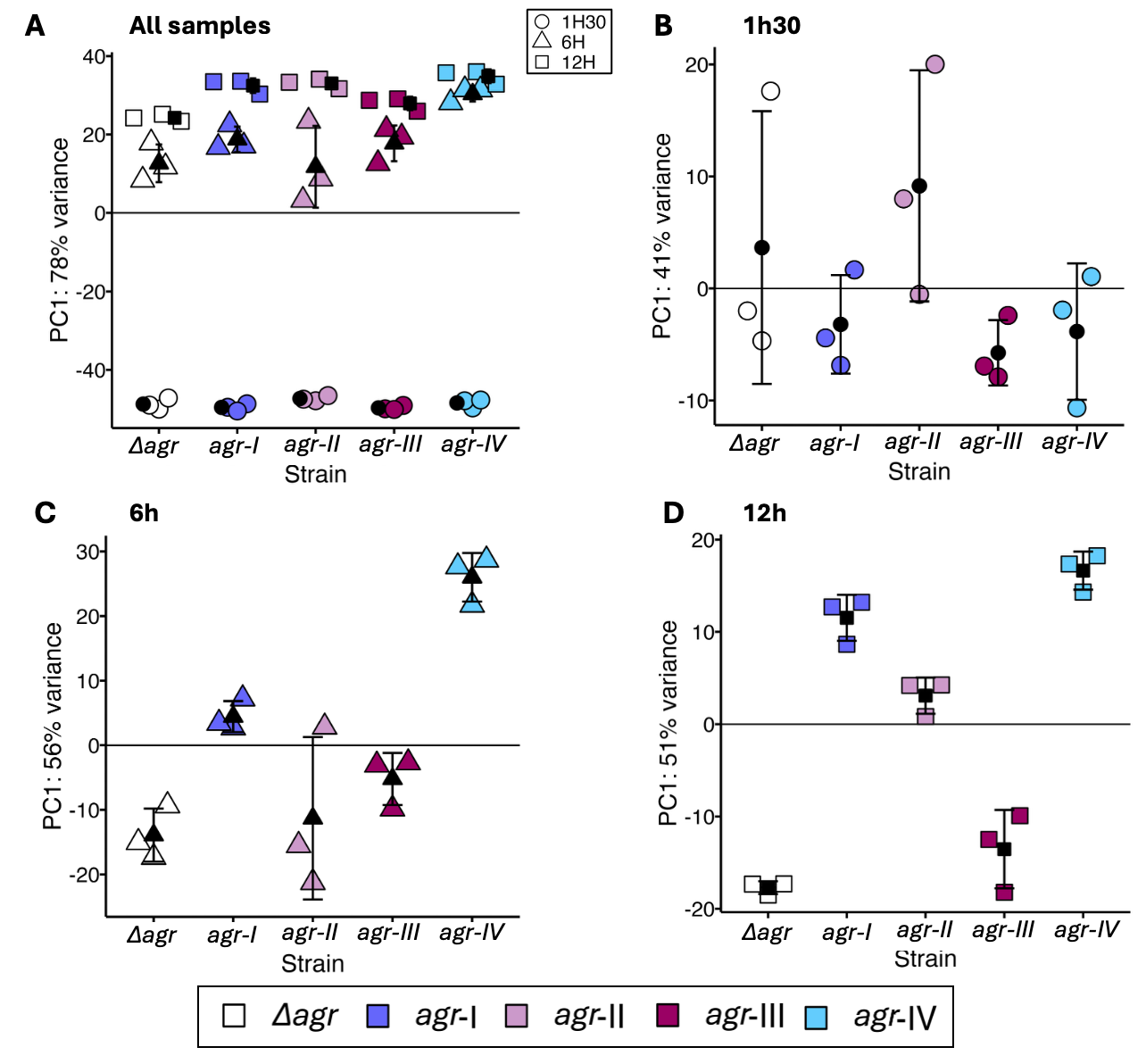

Supplement: S2 Fig — Dot-plots of the principal component analysis (PCA), using PC1 axis, depending on the agr-type, using the entire transcriptome beside the agr-locus, with all time points in A, at time point 1h30 in B, 6h in C and 12h in D. Standard error of the mean (SEM) for each time point in A, and per strain in B, C and D, are represented in black. (TIF) [file pone.0350108.s002.tif]
