# Supplementary material for: Staphylococcus aureus agr-type vs genetic background: molecular signatures determining differential metabolism and virulence potential
Source: PLoS One. 2026 Jun 3;21(6):e0350108. doi: 10.1371/journal.pone.0350108 (PMC13232812; doi:10.1371/journal.pone.0350108)
Supplement: Attachment — Submitted filename: Response to Reviewers_Final.pdf [file pone.0350108.s009.pdf]

We thank the Editor de Lencastre and the Reviewers for their careful reading of our manuscript and for their insightful comments and suggestions. We have revised the manuscript accordingly and believe that these changes have significantly improved the quality and clarity of the work. Below, we provide a detailed, point-by-point response to each comment. All changes in the revised manuscript are highlighted in red in the marked manuscript. Then, we mention additional modifications made in the manuscript to follow PLOS One authors guidelines.

Reviewer #1: The manuscript fills a significant gap in our understanding of S. aureus by isolating the effects of the agr system from its genetic background. Using congenic Newman strains, the authors demonstrate how different agr-types dictate the timing and intensity of transcriptional responses. The RNA-seq analysis across multiple growth stages offers valuable insights into the divergent regulation of virulence and metabolism.

The manuscript is well written and presents a robust experimental design. I present below a few minor comments that might improve the manuscript:

- In the 12h stationary phase, the authors note different enrichment in arginine biosynthesis. Given the importance of the arginine deiminase system (ADS) in pH homeostasis and survival, a more detailed analysis of the arc operon would strengthen the metabolism aspect of the title.

We thank Reviewer 1 for highlighting the importance of the ADS, and suggesting to include deeper discussion of its implication in our study. We now discuss a possible acidification of the medium and a switch in the carbon source used by the bacteria (line 211 to 215). In addition to the *arc* operon, the *kdp* and *ure* operons were also upregulated in *agr*-IV at 12h, although with reduced strength.

- The discussion regarding the ecological success of specific lineages remains somewhat speculative. While the congenic approach is a strength for isolating agr function, the "success" of a lineage in a clinical or environmental niche typically results from the co-adaptation of the agr type with its specific genetic background. The authors should explicitly discuss the limitations of using a single background (Newman) to draw broad ecological conclusions.

We thank Reviewer 1 of highlighting the co-adaptation of the *agr*-type with genetic background as a missing part in our discussion. We agree with Reviewer 1 and we attenuated the hypothesis of ecological success of given *agr*-lineages (line 216 to 219) and we explicitly mention the limitation of this study utilizing only one genetic background.

Reviewer #2: The present manuscript describes an impressive work on the role of the different agr-types over the expression of virulence factors and the overall modification of the transcriptome at three time points (1h30, 6h, and 12h).
The earlier and stronger activation in agr-IV has been previously described, but the authors eliminate the genetic background of the clones presenting the different agr-types to better study the transcriptomic differences associated to the sole variation on the agr system.
The text is clear, figures are easily understandable, and tables offer detailed information.

This reviewer only has a few comments to complete the manuscript:
1. Robustness: The data presented shows differences between agr-IV, and the rest of agr-types, particularly opposite to agr-III. But all experiments have been performed in triple replicates and to better support the observations more biological replicates seem necessary, particularly for

what is shown in Figure 2.
While the differences between agr-types are clear in the relative activation in Figure 1, the described three clusters are not so obvious in Figure 2.
Should the replicates not be feasible at this point, the authors should stress this weakness in the Discussion.

We thank the Reviewer 2 for raising this concern. Although more replicates would significantly increase the robustness of our results, we believe that the conclusions observed would remain unchanged, considering how biological replicates cluster together on the PC1 axis (Fig 2) and as observed in the new Supplementary Figure S2.

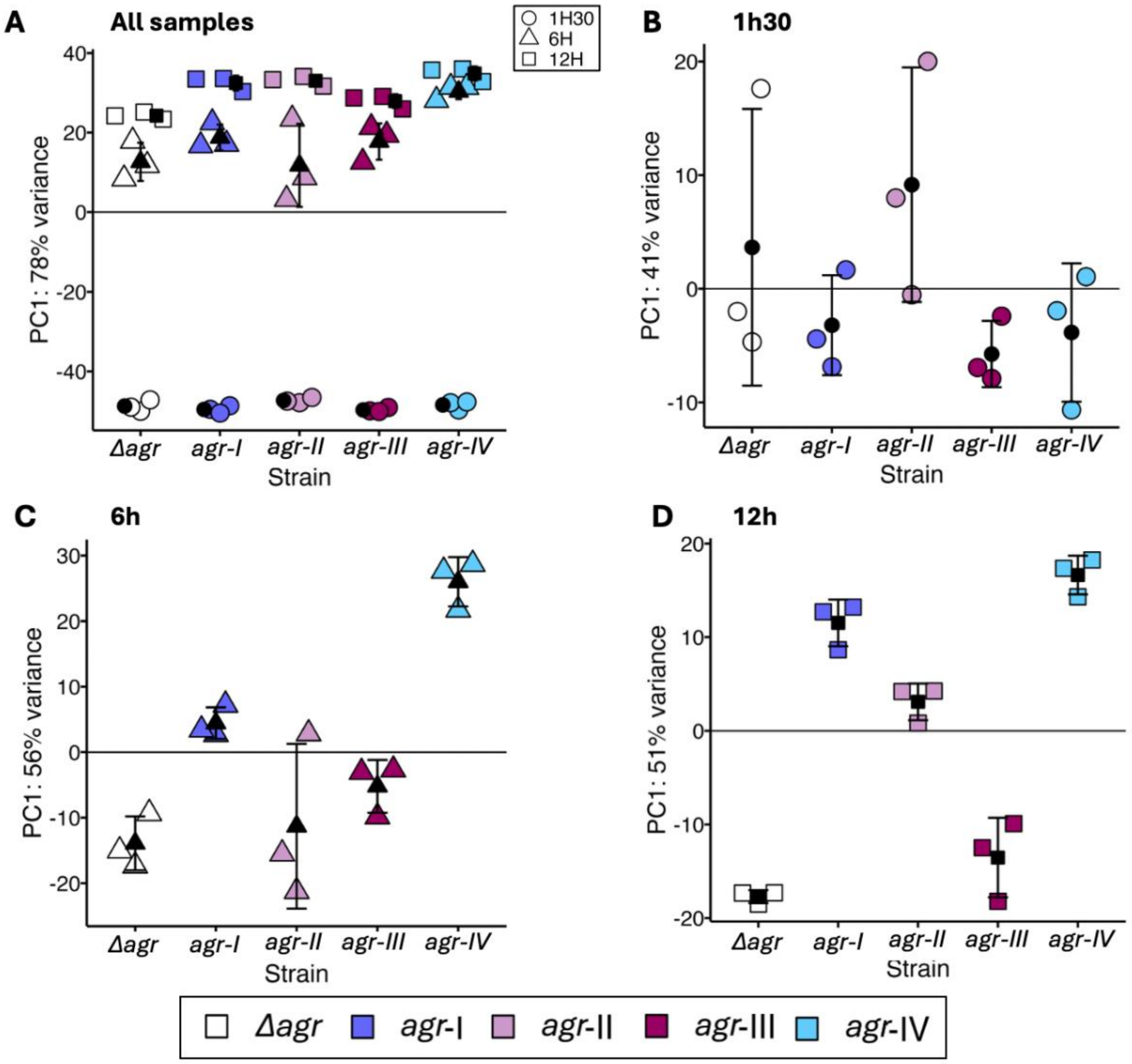

S2 Figure: Cluster identification from PCA analysis

Dot-plots of the principal component analysis (PCA), using PC1 axis, depending on the *agr*-type, using the entire transcriptome beside the *agr*-locus, with all time points in A, at time point 1h30 in B, 6h in C and 12h in D. Standard error of the mean (SEM) for each time point in A, and per strain in B, C and D, are represented in black.

Additionally, we have now emphasized how most clusters are observed focusing at the PC1 axis (Fig 2 and S2 Fig), which explains 56 and 51% of the variance at time points 6h and 12h, respectively, compared to only 18 and 10% for the PC2 axis (lines 110 to 112 in the Result section). In addition, we highlight now the variability observed within the clusters, line 115. We also discuss now in the Discussion section the limitation of performing only 3 replicates in this study, lines 216 to 219.

2. In-depth comparison: The authors describe well all the factors that are dramatically highly transcribed in agr-IV isolates when compared to the other agr-types. But further discussion, if any, is missing for the opposite behaviour regarding important S. aureus infection virulence factors such as protein A (spa -surprisingly lower in agr-IV than in any other agr-type), fibronectin-adhering protein (fnbA), chemotaxis-inhibiting protein (chp), which, like the cited superantigens (ssl group) are less expressed in agr-IV than in agr-II or agr-III. A comment on the putative virulence role of these differences is missing.

We appreciate the comment raised by Reviewer 2 regarding specific virulence factors. We added more detailed interpretation of our results in the Discussion section. Notably, we suggest that the downregulation of *spa* and *fnbA* in *agr*-IV is due to a faster activation of the *agr*-system, which negatively regulates these adhesion factors, and how it can contribute to *agr*-III virulence, lines 196 to 198. We also note the unexpected observation regarding *clfA*, lines 201 to 203. Finally, we discussed in more details the observation regarding *chp* in *agr*-IV at 6h, line 203 to 206.

3. Clinical significance: It could be interesting to address the probable role of the different transcriptome for the different agr-types in the pathogenesis in humans, for which the following citation is recommended, where early activation of agr-IV pathways is already described, and the importance of agr-III in menstrual toxic shock syndrome is highlighted. Jarraud S, Lyon GJ, Figueiredo AM, Lina G, Vandenesch F, Etienne J, Muir TW, Novick RP. Exfoliatin-producing strains define a fourth agr specificity group in Staphylococcus aureus. J Bacteriol. 2000 Nov;182(22):6517-22. doi: 10.1128/JB.182.22.6517-6522.2000. Erratum in: J Bacteriol. 2011 Dec;193(24):7027. Gérard, L [corrected to Lina, G]. PMID: 11053400; PMCID: PMC94802.

We thank Reviewer 2 for the suggestion and the reference. We added in the Discussion section that the early activation of *agr*-IV was previously observed, lines 207 to 208. Regarding the link between *agr*-types and *S. aureus* pathogenesis, we mention it in the Introduction, line 57: "Previous studies reported associations between agr-type and infection severity [7–9].". We now discussed it further in the Discussion section, line 221 to 225.

We would like to bring to the Reviewers and Editor's attentions that we have added 11 references to support newly discussed points in the Discussion section.

We also added in the Materials and Methods a new subsection: "Artificial Intelligence Tools and Technologies", detailing the use of AI for this manuscript.

We also updated the R script used for the statistical analysis, which includes the part added to generate graphs for the S2 Figure, and now mentions it in the subsection “RNAseq and Statistical Analysis” of the Materials and Methods section, instead of the Data Availability Statement section.
